# Accumulating Heterozygous Deleterious Mutations in Conserved Soybean Germplasm over Successive Regenerations

**DOI:** 10.3390/plants14152429

**Published:** 2025-08-05

**Authors:** Yong-Bi Fu, Carolee Horbach

**Affiliations:** Plant Gene Resources of Canada, Saskatoon Research and Development Centre, Agriculture and Agri-Food Canada, 107 Science Place, Saskatoon, SK S7N 0X2, Canada; carolee.horbach@agr.gc.ca

**Keywords:** deleterious mutation, mutation accumulation, mutation burden, plant germplasm conservation, germplasm regeneration, genebank, RIN, RNA-Seq

## Abstract

More than 5.9 million plant germplasm accessions currently conserved in over 850 national genebanks worldwide will accumulate deleterious mutations over long-term conservation. However, little is known about how mutations accumulate in germplasm under long-term conservation. An attempt was made using seed-based RNA-Seq analysis to identify and characterize deleterious genetic variants in 190 diverse soybean accessions that were conserved since 1972 and were regenerated up to 10 cycles. The analysis identified 588 deleterious variants, which were widely distributed across 20 soybean chromosomes, mostly present in 10 or fewer samples, associated with diverse biological processes, and largely predicted to be weakly and mildly detrimental. Significant differences in estimates of three mutation burdens (total, heterozygous, and homozygous) were found among the samples, including sample groups representing different countries of origin. Total and heterozygous mutation burden estimates were found to increase significantly with the number of conservation years since accession acquisition and the number of germplasm regenerations, but homozygous mutation burden estimates were not correlated with these two conservation-related accession features. Total mutation burden estimates were negatively correlated with expressed gene counts and RNA integrity numbers (RINs) and marginally positively associated with averaged gene expression levels. Correlations were also found among expressed gene count, averaged gene expression level, and RIN value. No significant differences were detected between seed-based and leaf-based estimates of total mutation burden, expressed gene count, averaged expression level, and RIN. These findings provide the first empirical evidence that total mutation burden increased primarily through the accumulation of heterozygous, rather than homozygous, deleterious mutations over successive soybean germplasm regenerations. This insight is useful for conducting informative assessments of deleterious mutation accumulation and enhancing the management and conservation of plant germplasm.

## 1. Introduction

More than 5.9 million plant germplasm accessions are currently conserved in over 850 national genebanks worldwide [1,2] and will accumulate deleterious mutations over long-term germplasm conservation [3,4,5,6]. Deleterious mutations will occur in plant germplasm (usually seeds, plant tissues, or whole plants) conserved ex situ and in situ due to changes in DNA that disrupt normal gene function. These deleterious mutations can accumulate from the joint actions of selection and genetic drift, both before and during germplasm regeneration, as elegantly modeled and illustrated with simulations by Schoen et al. in 1998 [6]. Deleterious mutation accumulation over the long term will alter the original genetic background of conserved germplasm, potentially increasing its vulnerability to future environmental pressures by reducing reproductive success and survival [7,8,9,10]. This accumulative genetic change could compromise some objectives of the long-term germplasm conservation mission, such as minimizing genetic shift and reducing loss of the original genotypes [6,11]. Thus, proper genebank management procedures need to be developed to minimize the genetic changes from deleterious mutation accumulation [11]. However, developing such mitigating strategies requires knowledge of how, and to what extent, deleterious mutations accumulate in germplasm conserved in genebanks, which is largely lacking.

Assessing mutation burden (or the extent of deleterious mutations) is technically possible and practically feasible, thanks to the advances in genomics, particularly from genetic load studies in the human genome (e.g., [12,13]) and bioinformatics tools developed for predicting deleterious amino acid polymorphism (e.g., [14]). Technically, genetic variants are screened across a sequenced genome and predicted to be deleterious mainly based on the gene function prediction of a nonsynonymous site change alone and/or in combination with the intensity of purifying selection inferred from phylogenetic restraints on the site. Over the last decade, increased efforts have been made to screen and report genome-wide deleterious genetic variants present in many plant genomes [15,16,17,18,19,20,21,22,23,24]. These predicted deleterious mutations are likely to be harmful to biological functions [9,23,25], although empirical evaluations of their overall effects on plant fitness are largely lacking [26]. Overall, these efforts have successfully demonstrated the usefulness of screening and characterizing deleterious genetic variants across plant genomes and provided informative estimations of mutation burden in plant germplasm [8,19,23,24].

Plant Gene Resources of Canada (PGRC; the Canadian national seed genebank at Saskatoon) maintains a soybean (*Glycine max* (L.) Merr.) germplasm collection of 1031 accessions. These accessions were mainly collected from Canadian soybean breeding programs over the last 50 years, and were acquired from the USDA-ARS soybean collection and the N.I. Vavilov All-Russian Institute of Plant Genetic Resources over the last 15 years as accessions with known early maturity. Since 2017, several studies have been conducted to characterize the PGRC soybean collection, generating the first comprehensive set of characterization data on maturity, oil and protein content, genetic distinctness, and mutation burden [22,27,28]. For example, one study detected a wide range of variation among the PGRC soybean accessions in each assessed trait. Based on these findings, four core subsets of 35 PGRC soybean accessions were developed, specifically targeting early maturity for cultivation in Saskatoon and Ottawa, and high oil and protein content. These findings are useful for the management and utilization of conserved soybean germplasm and are timely for enhancing Canadian soybean breeding, as soybean has emerged as the third largest field cash crop in Canada, with production expanding into the Canadian Prairies [29,30].

A recent study using leaf-based RNA-Seq [31] analysis of deleterious mutations in 70 accessions of the PGRC soybean collection identified 749 deleterious single nucleotide polymorphisms (SNPs) distributed across 20 soybean chromosomes and revealed a range of sample wise total mutation burden per deleterious locus from 0.204 to 0.268 with a mean of 0.232 and found that total mutation burden estimates increased with the years of conservation since accession acquisition [22]. However, little is known about whether these mutation findings are broadly applicable, as they could be specific to the early seedling stage of soybean development. Thus, this study was configured using seed-based RNA-Seq analysis with two major goals: (1) to assess the generality of the leaf-based mutation findings [22] and (2) to explore the correlations between mutation burden and conservation-related accession features. Specifically, 190 soybean accessions were selected, representing diverse countries of origin and characterized by three conservation-related features: (1) the number of conservation years since acquisition, (2) the number of conservation years since the last regeneration, and (3) the number of regenerations completed. As RNA analysis also allows for measuring RNA integrity number (RIN), counting expressed genes, and assessing averaged gene expression level at the sample level, efforts were also made to characterize these extra mutation-related estimates and assess their informativeness in analyzing deleterious mutations. The specific objectives of this seed-based study were to (1) measure RIN, identify deleterious variants, estimate mutation burden and expressed genes, and assess gene expression level for the 190 assayed accessions; (2) associate mutation burden and expressed gene and RIN estimates with the three conservation-related features; and (3) correlate seed-based and leaf-based estimates of mutation burden, expressed genes, and RIN. It was our hope that this study would allow for a better understanding of deleterious mutation accumulation in plant germplasm conserved in a genebank and generate useful findings to inform the development of effective management procedures to minimize genetic changes in conserved germplasm.

## 2. Materials and Methods

### 2.1. Sample Selection and Acquisition

We selected 190 soybean accessions (Table S1) from the PGRC soybean collection based primarily on four factors: variation in regeneration cycles, diversity of country of origin, inclusion in a previous related study [22], and seed availability for distribution. The selected accessions were acquired by PGRC from 1972 to 2004, were conserved in sealed envelopes with an assumed 20% relative air humidity under PGRC long-term storage at −18 °C following FAO Genebank Standards [32,33], and had gone through 0 to 10 regeneration cycles since acquisition. These accessions consisted of landraces, cultivars, and breeding lines. They also represented 21 countries, with each country contributing between 1 and 69 accessions. Among the 190 accessions, 66 were previously assayed using leaf-based RNA-Seq analysis, which allowed for comparisons between seed-based and leaf-based estimates of mutation burden. Soybean seed samples and their inventory data (Table S1), such as passport, country of origin, year of acquisition, year of the last regeneration, and number of regenerations, were acquired in September 2022 from Ms. Colleen Nielson at PGRC for public good research, following the Standard Material Transfer Agreement of the International Treaty on Plant Genetic Resources for Food and Agriculture (https://www.fao.org/plant-treaty/areas-of-work/the-multilateral-system/smta/en/; accessed 30 July 2025).

### 2.2. RNA-Seq Analysis

For each accession, a single dry seed was randomly selected. Total RNA was extracted from the seed embryo using an RNeasy Plant Mini Kit (Qiagen Inc., Toronto, ON, Canada) with buffer RLT, following the manufacturer’s protocol. Briefly, single embryos were individually placed in 2 mL microcentrifuge tubes, flash-frozen in liquid nitrogen, and ground using a 5 mm steel bead in a TissueLyser II (Qiagen Inc., Toronto, ON, Canada) at 20 Hz for 2.5 min. The TissueLyser Adapter Set (2 × 24) also from Qiagen was precooled at −80 °C for at least 2 h prior to use [34]. To minimize guanidine thiocyanate carryover, the final wash with buffer RPE was repeated. Following extraction, RNA samples were treated using an Invitrogen™ DNA-free™ Kit (ThermoFisher Scientific, Waltham, MA, USA) to eliminate genomic DNA contamination. RNA was quantified using a Thermo Scientific Nanodrop 8000, and RIN for each sample was measured using an RNA 6000 Nano Kit on an Agilent 2100 Bioanalyzer (Agilent Technologies, Santa Clara, CA, USA). The RIN values are provided in Table S2. RNA samples were stored at −80 °C until library preparation. mRNA-Seq libraries were prepared using a Lexogen CORALL mRNA-Seq Library Prep Kit V2 with Poly(A) Selection (Lexogen Inc., Greenland, NH, USA), following the manufacturer’s protocol for long insert sizes (RTL Chemical, Huzhou, Zhejiang, China). Libraries were indexed using unique dual indexes (UDI) with built-in unique molecular identifiers (UMI), which were included in the library preparation kit. The indexed libraries were pooled and sequenced at the Centre d’expertise et de services Génome Québec on a single lane of an Illumina NovaSeq 6000 S4 flow cell, generating 100 bp paired-end reads. The acquired raw sequences were deposited in 2024 to the National Center for Biotechnology Information Sequence Read Archive database under the BioProject ID PRJNA1140562.

### 2.3. SNP Calling

RNA sequencing generated a pair of demultiplexed forward and reverse FASTQ files for each sample. FastQC v0.12.0 [35] was used to assess the overall sequencing quality of each sample. The first 12 bases of each FASTQ R1 sequence contained a UMI, which was incorporated during library preparation. To remove the UMIs, FASTQ R1 sequences were trimmed using Trimmomatic v0.39 [36] with the setting HEADCROP:12. The FASTQ R1 files were then trimmed further, along with the FASTQ R2 files, using Trimmomatic v0.39 to remove any adapter sequences, trim low-quality sequences (below a Phred score of 24), and remove any sequences shorter than 80 bases. The following trim settings were used: ILLUMINACLIP: TruSeq3-PE-2.fa; SLIDINGWINDOW:10:24; and MINLEN:80. FastQC was re-run after trimming to verify the removal of the Illumina adapter sequences. The soybean genome assembly of Wm82.a2.v1 [37,38] was used in this study. The sample FASTQ files were aligned against the reference soybean genome sequence using the Burrows–Wheeler Aligner v0.7.17 [39] BWA-MEM algorithm. The resulting BAM files were filtered to remove PCR duplicates using the MarkDuplicates tool from the Genome Analysis Toolkit v4.2.6.1 [40]. Samtools sort was applied to produce sorted BAM files. SNP calling without indels was performed using Bcftools v1.9 [41] with the following command: bcftools mpileup -Ou -f -b | bcftools call -vmO z -V indels -o. SNP quality filtering was performed using Vcftools v0.1.15 [42] with the following command: vcftools --vcf input.vcf --out output.vcf --recode-INFO-all --max-alleles 2 --min-alleles 2 --minDP 10 --minQ 20 --max-missing 1 --recode.

### 2.4. Identification of Deleterious SNPs

SNP annotation was performed using the stand-alone Ensembl Variant Effect Predictor (VEP) v96 [43,44] from the generated SNP VCF file. The sorting intolerant from tolerant (SIFT) algorithm [45] was applied to predict the deleterious effect of a genetic variant on its gene function, and each SNP was annotated with a SIFT score. The SIFT score measures the predictive impact of an amino acid substitution and can distinguish between functionally neutral and deleterious amino acid changes. An amino acid substitution with a SIFT score of 0.05 or less is considered to be deleterious. The SIFT analysis was performed based on a previously generated soybean SIFT database [22]. To increase the accuracy of identifying deleterious SNPs (dSNPs), this study applied both the SIFT score and GERP++ Rejected Substitution (RS) score [46] to evaluate a SNP. The RS scores for the extremely conserved chromosomal regions of the soybean genome were previously generated using GERP++ based on the reference genomes of 12 plant species to measure the phylogenetic constraint from the substitution of a locus [22]. The resulting RS score provides a quantification of the conservation of each nucleotide in a multi-species alignment. A positive score (RS > 0) at a substitution site means fewer substitutions than expected. Thus, a substitution occurring in a conserved site with RS > 0 is predicted to be deleterious; the larger the RS score, the more deleterious the substitution. Specifically for this study, SIFT (<0.05) and GERP++ RS (>0) annotations were combined to identify dSNPs in constrained portions of the genome. The identified dSNPs were further categorized as weakly, mildly, and highly detrimental based on their GERP++RS scores: <1, 1–3, and >3, respectively. Note that a dSNP (or mutation) in this study is defined as a SNP (or mutation) predicted to be deleterious to its gene function, not necessarily to overall plant fitness. Based on the previous studies [9,23,25], the genetic variants identified with the current approach are more likely to be detrimental to biological functions.

Based on the original deleterious SNP genotype VCF file generated from this study, minor allelic frequencies in the assayed samples were analyzed using Vcftools, and fixed dSNPs were identified based on the allelic frequency data for all assayed samples. The dSNPs and total detected SNPs were also counted for each chromosome to compare their genomic distributions.

### 2.5. Gene Ontology (GO) and Expression Analysis

GO analysis of the predicted dSNPs was performed first with the identification and extraction of genes associated with the identified dSNPs from the soybean gene annotation file. The associated gene list was analyzed using ShinyGO v0.82 [47] to generate enriched GO terms and to identify enriched gene regions across the soybean chromosomes. The resulting GO term sets were further analyzed and visualized using REVIGO v1.8.1 [48] with treemaps and tag clouds to assist in the interpretation of gene enrichments and functions.

Expression analysis of the genes associated with the identified dSNPs was performed by first extracting the abundance of sequence reads for the associated genes and then identifying (1) all the expressed genes in all the assayed samples, along with their expression levels, and (2) specific expressed genes in each sample. This was performed using the RNA-Seq data and a custom shell script based on the StringTie program v1.3.4b [49]. This expression analysis generated two new data sets per sample (the expressed gene count and the averaged gene expression level), which are given in Table S2. It also identified all the associated genes expressed in the 190 assayed samples, which are given in Table S3.

### 2.6. Mutation Burden Estimation and Its Association with Conservation-Related Features

Mutation burden per deleterious locus for an individual sample was calculated from sample deleterious SNP genotype data based on the number of deleterious alleles [19]. Three burden models were considered: total mutation burden, heterozygous mutation burden, and homozygous mutation burden. The homozygous mutation burden per deleterious locus is the number of derived deleterious alleles in the homozygous state, divided by a product of 2 × total dSNP count. The heterozygous mutation burden per deleterious locus is the number of derived deleterious alleles existing in the heterozygous state, divided by a product of 2 × total dSNP count. The total mutation burden per deleterious locus is the number of derived deleterious alleles existing in an accession (2 × homozygous mutation burden + heterozygous mutation burden), divided by the product of 2 × total dSNP count. These three burdens per deleterious locus were estimated for each sample, and the resulting estimates are given in Table S2.

To characterize these burden estimates, the three estimates were plotted using the R barplot function to evaluate their distribution patterns. The analysis of variance was also performed on these three burden estimates with respect to country of origin to determine if differences in burden estimates exist among conserved accessions representing different countries. A linear regression analysis was conducted using the R lm function [50] of the estimates of a mutation burden per deleterious locus (total, heterozygous, or homozygous) over the three conservation-related sample features (the number of conservation years since the last accession regeneration, the number of conservation years since accession acquisition, and the number of regeneration cycles since accession acquisition). The results were plotted using the R plot function.

### 2.7. Additional Association Analyses

This study not only generated three mutation burden estimates per sample, but also the estimates of the expressed genes, their averaged expression level, and RIN, as described above. To understand these extra estimations and their relationship to burden estimates, the R barplot function was applied to display the distribution patterns of the estimates. Additionally, linear regression analysis was performed on each pair of these six estimates using the R lm function. Extra linear regression analyses were also performed on the estimates of expressed gene count, averaged expression level, and RIN over the three conservation-related sample features as described above.

### 2.8. Comparison of Seed-Based and Leaf-Based RNA-Seq Analyses

This seed-based RNA-Seq analysis shared 66 accessions with the previous leaf-based RNA-Seq analysis [22], allowing for comparisons of various estimations. Thus, pairwise correlations among three burden estimates, two estimates of expressed genes, and RIN values in these 66 samples were examined using linear regression analyses. These correlations will enable a clearer comparison of the informativeness of the estimations. Extra effort was also made to find the shared deleterious variants identified by both analyses, with the results presented in Table S4.

## 3. Results

### 3.1. SNP Identification and Annotation

RNA-Seq analysis produced a total of 2643 million sequence reads for 190 soybean samples with an average of 13.6 million mapped sequence reads per sample. SNP calling without indels from the RNA-Seq data identified 58,548 SNPs without missing values (Table 1). These identified SNPs were widely distributed across 20 soybean chromosomes (Figure S1A). Specifically, the SNP count per chromosome ranged from 2341 to 4029, with an average of 2927.4. These SNPs displayed expected L-shape distributions of minor allele frequency (Figure S2A). A majority (56.2%) of the SNPs had low frequencies of 0.05 or smaller, but there were 1344 (or 2.3%) invariant heterozygous SNPs with a minor allele frequency of 0.5.

VEP-based annotation analyses of the identified SNPs allowed for the classification of SNPs into 16 different classes with the most severe consequences (Table 1). The classes with the most SNPs were synonymous_variant (20,478), missense_variant (19,847), 3_primer_UTR_variant (7068), and 5_primer_UTR_variant (5354). The proportions of synonymous_variant and missense_variants over all the identified SNPs were 0.350 and 0.339, respectively. There were 869 (or 1.5%) loss-of-function SNPs, consisting of stop_gained, stop_lost, stop_retained, splice_acceptor, splice_donor, splice_region, and start_lost variants (Table 1).

### 3.2. Deleterious Mutation

Screening SIFT scores across all the non-synonymous SNPs revealed 6853 deleterious SNPs. After excluding 941 deleterious_low_confidence SNPs, the dSNPs were reduced to 5912, accounting for 10.1% of all identified SNPs (Table 1). Combining SIFT scores with RS scores identified 588 SNPs as deleterious, representing 1% of the identified SNPs (Table 1). Also, one deleterious SNP was found to be fixed in all assayed samples. These 588 dSNPs were widely distributed across 20 soybean chromosomes (Figure S1B). Specifically, the SNP count per chromosome ranged from 8 (Chromosome 18) to 63 (Chromosome 10) with an average of 29.4. The dSNPs also displayed expected L-shape distributions of minor allele frequency (Figure S2B). Specifically, there were 507 (86.2%) dSNPs with minor allelic frequencies of 0.05 or less (or present in 10 or fewer samples) and 123 (20.9%) with 0.01 or less (or present in one sample only). Thus, most of the identified dSNPs were located in only a few soybean samples. The identified dSNPs could be categorized as weakly, mildly, and highly detrimental based on their GERP++ RS scores: <1, 1–3, and >3, respectively (Table 1; Figure S1C). Specifically, there were 45 (7.7%) weakly, 477 (81.1%) mildly, and 66 (11.2%) highly deleterious SNPs. Thus, a large majority (88%) of the 588 dSNPs were weakly and mildly detrimental.

### 3.3. Ontology of the Associated Genes

The identified dSNPs were found to be associated with 773 soybean genes across all chromosomes (Figure S3). The ontology analyses of the associated genes using ShinyGO identified 137 significant (*P* < 0.05) GO terms. Analysis of these GO terms using REVIGO revealed 52 biological processes, 42 cellular components, and 27 molecular functions for the associated genes (Figure S4). The major biological processes were RNA splicing via transesterification reactions with bulged adenosine as nucleophile, cellular metabolic process, regulation of translation, protein-containing complex organization, cellular process, metabolic process, peptide metabolic process, and amide metabolic process. The major cellular components were spliceosomal complex, cytoplasm, intracellular anatomical structure, organelle, cellular anatomical entity, and protein-containing complex. The major molecular functions were thiamine pyrophosphate binding, phosphatase activity, small molecular binding, catalytic activity acting on a protein, catalytic activity, binding, RNA helicase activity, and glycerol-3-phosphate O-acyltransferase activity. As expected for stored seeds, these associated genes were mainly involved with RNA splicing, related cellular components, and molecular binding.

Extracting the sequence reads using the StringTie program for the 773 associated genes in each sample of the RNA-Seq data revealed a total of 175 canonical associated genes that were expressed in 1 to 190 assayed seed samples (Table S3). Specifically, there were 119 genes expressed in all the samples, 156 genes in 107 or more samples, 19 genes in 89 or fewer samples, and 8 genes in 18 or fewer samples. These expressed genes were widely distributed across 19 chromosomes but absent in Chromosome 14. Extra GO analysis of the 175 expressed genes revealed significant GO terms associated with only two molecular functions: mRNA binding and RNA binding.

### 3.4. Mutation Burden

Estimates of three mutation burdens (total, heterozygous, and homozygous) per deleterious locus were made per sample, as shown in Figure S5A–C. Clearly, there were large variations in the three mutation burden estimates (Table S2). The estimates of total individual mutation burden ranged from 0.033 to 0.068, with a mean of 0.051 and a standard deviation of 0.007. Specifically, the five accessions with the highest total mutation burden estimates were CN35370, KAS642/7 from South Korea (0.068); CN32766, No. 601 from Poland (0.068); CN35264, Ajma from Poland (0.066); CN107573, Voronyezskaja from Russia (0.065); and CN107468, B/15 (821) from Lithuania (0.065). The five accessions with the lowest total mutation burden estimates were CN33273, Beechwood from Canada (0.0374); CN52650, Krapinka from Russia (0.0366); CN107419, Alex from Canada (0.036); CN107425, Accord from Canada (0.035); and CN52640, Amurskay 310 from Russia (0.033). The estimates of heterozygous mutation burden ranged from 0.005 (CN107418, B0501 from Canada; CN107425, Accord from Canada; CN107459, National from Canada) to 0.031 (CN32766, No.601 from Poland) with a mean of 0.015 and a standard deviation of 0.005. Similarly, the estimates of homozygous mutation burden ranged from 0.020 (CN107854, 744/2 from Sweden) to 0.055 (CN39215, X1590 from Canada) with a mean of 0.036 and a standard deviation of 0.007.

Analysis of variance of the three mutation burden estimates among 17 groups of samples representing different countries revealed significant differences in total mutation burden and heterozygous mutation burden estimates (Figure S6), but not in homozygous mutation burden estimates. The means of total mutation burden estimates ranged from 0.0454 (Hungary) and 0.0470 (Yugoslavia) to 0.0590 (Switzerland) and 0.0627 (Poland) (Figure S6A) and the means of heterozygous mutation burden estimates ranged from 0.0127 (Yugoslavia) and 0.0129 (Canada) to 0.0230 (Switzerland) and 0.0237 (Poland) (Figure S6B). Thus, significant differences in these two burden estimates existed among samples originating from the European countries, suggesting that the sample origins were associated with the estimates of total mutation burden.

### 3.5. Associations Between Mutation Burdens and Sample Features

**Figure 1:**
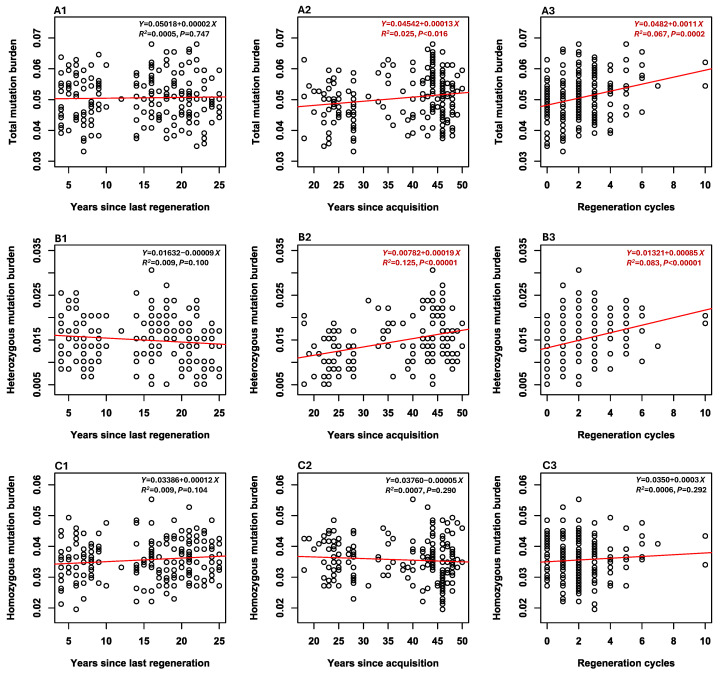
Associations of the estimates of three mutation burdens (total (**A1**–**A3**), heterozygous (**B1**–**B3**), and homozygous (**C1**–**C3**)) with three conservation-related accession features (the number of conservation years since the last accession regeneration (1), the number of conservation years since accession acquisition (2), and the number of regeneration cycles (3)). Linear regression line in each panel is shown in red. The significant associations highlighted in red indicate that heterozygous mutation burden, not homozygous mutation burden, was accumulated in conserved germplasm since accession acquisition over successive regenerations.

The linear regression analyses of three mutation burden estimates per sample (total, heterozygous, and homozygous) over the three conservation-related features revealed four (out of nine) significant associations (Figure 1). Specifically, the total mutation burden estimates of the 190 soybean samples significantly increased with the number of conservation years since accession acquisition (Figure 1(A2)) and the number of regeneration cycles since accession acquisition (Figure 1(A3)). Increases were also found with these two conservation-related features for heterozygous mutation burden estimates (Figure 1(B2,B3)). Both total and heterozygous mutation burden estimates were not associated with the number of conservation years since the last regeneration (Figure 1(A1,B1), respectively). Interestingly, homozygous mutation burden estimates were not associated with any of these three conservation-related features (Figure 1C).

### 3.6. Three Other Mutation Estimates

This study also generated three extra mutation-related estimates per sample (expressed gene count, averaged gene expression level, and RIN; Table S2). Large variations among the 90 samples were observed for these three estimates. The number of the expressed canonical genes per sample ranged from 130 (CN107420, AC 2001 from Canada) to 164 (CN42536, Dono 36 from China; CN107563, Wielnska Brunatna from Hungary), with an average of 155 (Figure S5D). Averaging the transcripts per million (TPM) per gene across all of the expressed genes in a sample revealed a range of 9.88 (CN107357, RCAT Angora from Canada) to 21.04 (CN35264, Ajma from Poland), with an average of 13.16 (Figure S5E). RIN values ranged from 6.5 to 9.6 with an average of 7.7 (Figure S5F).

Extra effort was made to assess the differences in these three estimates among the samples representing different countries. Significant differences were found only in the averaged gene expression levels among the samples originating from different countries (Figure S6C). The two accessions with the highest averaged expression TPMs were from Hungary (14.99) and Poland (14.89), and the two accessions with the lowest averaged expression TPMs were from South Korea (12.14) and Sweden (11.98). These results suggested that the estimates of expressed gene count and RIN were not associated with the sample origins.

### 3.7. Associations Between Mutation Burdens and Other Mutation Estimates

**Figure 2:**
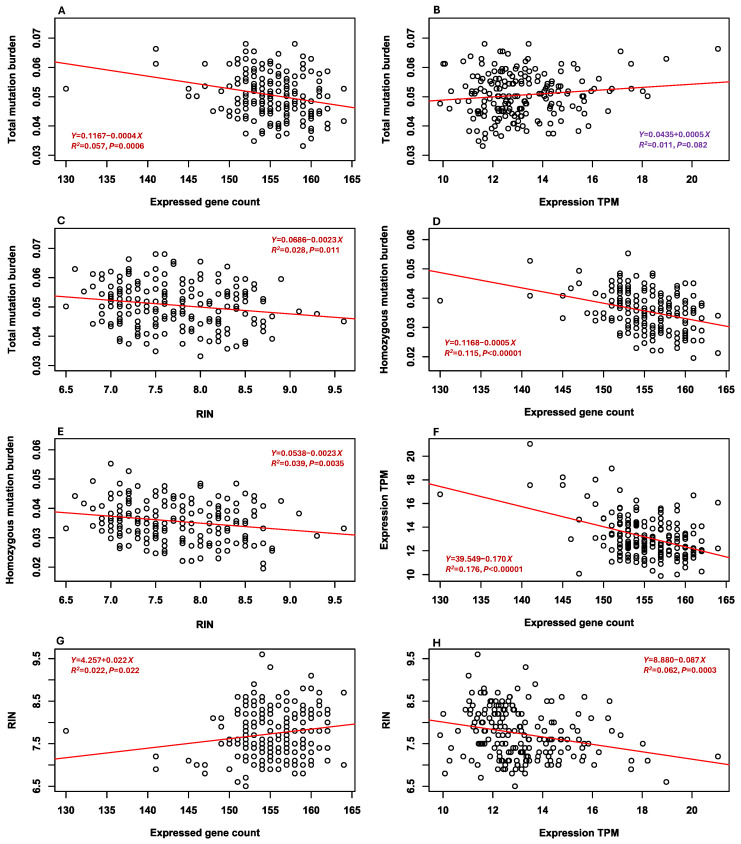
Eight significant and marginally significant associations detected among the estimates of three mutation burdens (total, heterozygous, and homozygous), expressed gene count, averaged expression level of the associated genes, and RIN, as illustrated in panels (**A**–**H**). Linear regression line in each panel is shown in red. The marginally significant association in the panel (**B**) is highlighted in purple.

The pairwise correlation analyses of six estimates in the 190 samples revealed seven significant and one marginally significant association (Figure 2). Specifically, the estimates of total mutation burden were negatively associated with expressed gene counts (Figure 2A) and RIN measures (Figure 2C), and marginally positively correlated with averaged gene expression levels (Figure 2B). The estimates of homozygous mutation burden were negatively correlated with expressed gene counts (Figure 2D) and RIN values (Figure 2E). The estimates of expressed gene count were negatively associated with the averaged gene expression levels (Figure 2F). RIN values were positively associated with the expressed gene counts (Figure 2G) but negatively correlated with the averaged gene expression levels (Figure 2H). Interestingly, the estimates of heterozygous mutation burden were not correlated with expressed gene counts, the averaged gene expression levels, or RIN values.

### 3.8. Associations Between Three Other Mutation Estimates and Sample Features

Extra effort was made to find the correlations of expressed gene counts, averaged gene expression levels, and RIN values with the three conservation-related features of the accessions. It was found that expressed gene counts increased only with the number of conservation years since accession acquisition (Figure S7(A2)). The averaged gene expression levels were positively associated with the number of conservation years since the last regeneration (Figure S7(B1)) and negatively correlated with the number of regeneration cycles since accession acquisition (Figure S7(B3)). Also, RIN values decreased significantly only with the number of conservation years since the last regeneration (Figure S7(C1)), but not with the other two conservation-related features (Figure S7(C2,C3)).

### 3.9. Comparisons Between Seed-Based and Leaf-Based Estimates

**Figure 3:**
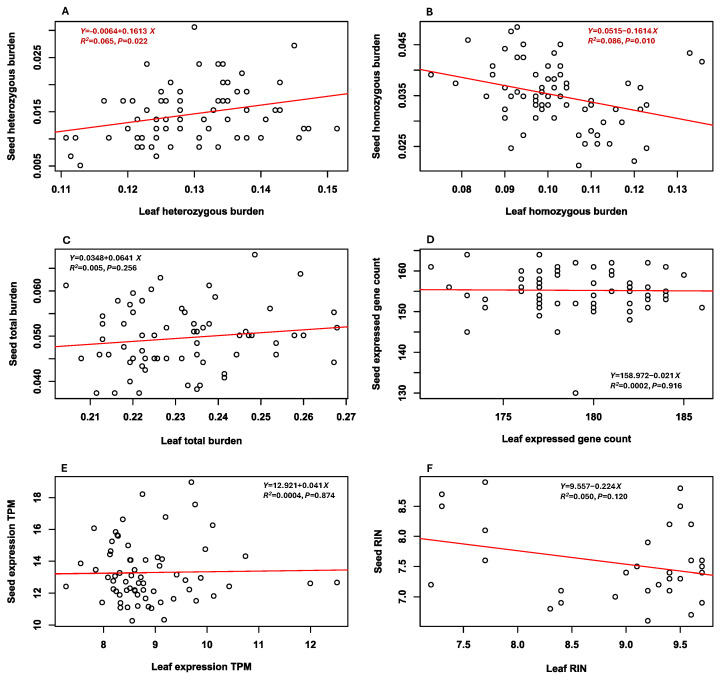
Pairwise correlations between the seed-based and leaf-based estimates of heterozygous mutation burden (**A**), homozygous mutation burden (**B**), total mutation burden (**C**), expressed gene count (**D**), averaged expression level of the associated genes (**E**), and RIN in 66 assayed soybean accessions (**F**). Linear regression line in each panel is shown in red. Two correlations (**A**,**B**) highlighted in red were statistically significant, while the other four correlations (**C**–**F**) were not statistically significant at *P* < 0.05, indicating that the seed-based and leaf-based RNA-Seq analyses yielded similar estimates of total mutation burden and expressed genes.

Pairwise correlation analyses of six estimates generated from 66 samples in the two different RNA-Seq data sets revealed two significant associations (Figure 3). Seed-based heterozygous mutation burden estimates increased with the higher leaf-based heterozygous mutation burden estimates (Figure 3A). Seed-based homozygous mutation burden estimates decreased with the higher leaf-based homozygous mutation burden estimates (Figure 3B). Seed-based total mutation burden estimates showed only the trend (*P* = 0.256) of increasing with the higher leaf-based total mutation burden estimates (Figure 3C). Similarly, no correlations were observed between the two RNA-Seq data sets in expressed gene count, averaged gene expression level, or RIN value (Figure 3D–F). Thus, there were no significant differences in total mutation burden, expressed gene count, averaged expression level, or RIN value between the two RNA-Seq data sets.

This seed-based analysis identified 588 deleterious SNPs in the 190 samples, while the previous leaf-based analysis identified 749 deleterious SNPs in the 70 samples. Interestingly, there were 72 deleterious SNPs shared between these two analyses (Table S4). The shared deleterious SNPs were widely distributed across 16 chromosomes, but absent in chromosomes 11, 14, 18, and 20. Two shared deleterious SNPs with GERP++ RS scores of 3 or higher were predicted to be highly deleterious, fifty-five were mildly deleterious with RS = 1–3, and fifteen were weakly deleterious with RS < 1.

## 4. Discussion

This seed-based RNA-Seq analysis generated a set of novel findings on deleterious mutation accumulations in soybean germplasm that was conserved in a genebank since 1972. First, the analysis identified 588 deleterious SNPs (Table 1) that were widely distributed across the 20 chromosomes (Figure S1B), mostly present in only a few samples (Figure S2B), associated with diverse biological processes (Figure S4), and largely predicted to be weakly and mildly detrimental (Figure S1C). Second, significant differences in the three mutation burden estimates were observed among samples, including the sample groups representing different countries of origin (Figure S6). Total mutation burden and heterozygous mutation burden estimates increased significantly with the number of conservation years since accession acquisition and the number of regeneration cycles, but homozygous mutation burden estimates were not associated with these two conservation-related accession features (Figure 1). Third, total mutation burden estimates were negatively associated with expressed gene counts and RIN values and marginally positively correlated with averaged gene expression levels (Figure 2). Correlations were also found among expressed gene count, averaged gene expression level, and RIN value. Fourth, no significant differences were detected between seed-based and leaf-based estimates of total mutation burden, expressed gene count, averaged expression level, and RIN (Figure 3). These findings are significant, as they provide the first empirical evidence that total mutation burden increased primarily through the accumulation of heterozygous, rather than homozygous, deleterious mutations over successive germplasm regenerations. They are useful for informative assessments of deleterious mutation accumulations and for improving genebank management procedures aimed at minimizing genetic changes in conserved germplasm.

The seed-based mutation burden analysis not only confirmed the previous leaf-based mutation burden finding (see Figure 2A of Fu et al. [22]) that total mutation burden estimates increased with the number of conservation years since accession acquisition (Figure 1(A2)), but also revealed an important finding that total mutation burden estimates also increased with up to 10 regeneration cycles (Figure 1(A3)), but not with the number of conservation years since the last regeneration (Figure 1(A1)). Interestingly, the heterozygous mutation burden estimates, like the total mutation burden estimates, also increased with both the number of conservation years since accession acquisition and the number of regeneration cycles, but not with the number of conservation years since the last regeneration (Figure 1B). More interestingly, however, the homozygous mutation burden estimates were not correlated with these three conservation-related features (Figure 1C). These findings clearly indicate that the total mutation burden was built up mainly through the accumulation of heterozygous, not homozygous, deleterious mutations over germplasm regenerations. Genetically, this explanation is reasonable: if homozygous deleterious mutations arise during cold storage, they are likely to be purged out through subsequent germplasm regenerations. The larger the deleterious effect of a mutation, the higher the chance it will be selected against, particularly with a small sample size used in germplasm regeneration [51,52]. In contrast, the heterozygous deleterious mutations are less likely to be eliminated from the germplasm in each cycle of germplasm regeneration [53], which allows them to accumulate over various regeneration cycles. Together, these findings provide clear-cut empirical evidence that accumulating heterozygous deleterious mutations over germplasm regenerations contributed more to the total mutation burden in conserved soybean germplasm than accumulating homozygous deleterious mutations.

Associating three mutation burden estimates with two expressed gene estimates (Figure 2) revealed only two significant correlations between total mutation burden and homozygous mutation burden estimates with expressed gene counts (Figure 2A,D) and one marginally significant correlation between total mutation burden estimates and averaged gene expression levels (Figure 2B). Interestingly, no associations were found between heterozygous mutation burden estimates and the two expressed gene estimates. These findings suggest that expressed gene count carried some information on deleterious mutations, particularly on homozygous deleterious mutations, while averaged gene expression level carried much less, although both expressed gene counts and averaged gene expression levels were negatively correlated (Figure 2F). Associating two expressed gene estimates with the three conservation-related accession features revealed (1) that expressed gene counts were positively correlated with the number of conservation years since accession acquisition (Figure S7(A2)), and (2) that averaged gene expression levels increased with the number of conservation years since the last regeneration (Figure S7(B1)) and decreased with the number of regeneration cycles (Figure S7(B3)). These two sets of research findings are novel and interesting, as these expressed genes represented only the genes that were associated with the deleterious SNPs, expressed before seed germination, and largely involved with RNA and mRNA bindings. However, the findings indicate that these two expressed gene estimates (Table S2; Figure S5D,E) were not sensitive to the assessment of deleterious mutations and carried relatively less information on deleterious mutations than the total mutation burden estimates.

Measuring RNA integrity is an emerging technique for predicting the longevity of seeds under long-term storage conditions [54,55,56,57,58,59]. It is based on assessing RNA quality in stored seeds by quantifying the extent of RNA fragmentation using RIN. In dry seeds, RIN appears to function as an indicator of aging, with its values corresponding to the rate of seed deterioration. Consequently, RIN-based prediction of seed longevity is technically feasible [55,56,58]. Our RNA analysis revealed a large variation of RIN values for the 190 assayed soybean samples (Table S2; Figure S5F). Interestingly, the RIN values decreased significantly with the number of conservation years (ranging from 4 to 25) since the last soybean germplasm regeneration (Figure S7(C1)) but were not correlated with the number of conservation years since germplasm acquisition (Figure S7(C2)) or the number of regeneration cycles (Figure S7(C3)). This finding is largely expected as RNA will degrade more with increased years of cold storage without regeneration. More interestingly, the RIN values increased significantly with increased expressed gene counts (Figure 2G), but with decreased total mutation burden estimates (Figure 2C), homozygous mutation burden estimates (Figure 2E), and averaged gene expression level (Figure 2H). Although these significant correlations explain only 2.2 to 6.2% of the observed variances, they clearly indicate that the RIN values were associated with deleterious mutations harbored in the conserved soybean germplasm. This association is further supported by the findings of the 175 expressed genes associated with the deleterious SNPs and their involvement with the molecular functions for RNA and mRNA binding. Further explorations of RIN and its association with deleterious mutations may inform the potential applications of RIN to predict deleterious mutations, particularly in association with the number of conservation years since the last regeneration, as it is much simpler to measure RIN values than to estimate mutation burden from sequence data.

The interesting finding of no significant differences between seed-based and leaf-based estimates of total mutation burden, expressed gene count, averaged expression level, and RIN (Figure 3) is useful for future RNA-Seq analyses assessing deleterious mutations in conserved germplasm, as seed-based and leaf-based data sets carried essentially similar information on mutation burden (Table S4). The only exception is that the seed-based data set carried more homozygous mutation burden than the leaf-based data set (Figure 3B). This exception is expected, as some homozygous deleterious mutations may be purged out through unsuccessful seed germination or failure to reach the seedling stage for the leaf-based RNA-Seq analysis. In contrast, the leaf-based heterozygous mutation burden estimates increased with the seed-based heterozygous mutation burden estimates (Figure 3A), as the heterozygous deleterious mutations were less likely to be eliminated during seed germination or seedling growth, as discussed above. Thus, our findings seem to favor the use of a seed-based approach for large-seeded plants such as soybean [59]. When RNA extraction is less feasible for smaller dry seeds, a leaf-based approach could still yield similar estimates of mutation burden. However, our seed-based and leaf-based comparisons here with only 66 sample pairs were preliminary, and more comparative research is preferred to have a comprehensive understanding of the differences in identification of deleterious mutations and estimation of mutation burden.

This study also carried some weaknesses worth mentioning. First, the RNA-Seq analysis assayed only a single seed per accession and did not consider the variability of deleterious mutations among seeds of each accession. However, such mutation variability should still exist within an accession of a selfing plant, as demonstrated in conserved barley germplasm [60]. Second, the mutation identification was dependent on many factors, including the quality of sequencing data, assembled reference genome, bioinformatic tools used for mutation screening, and sample size. Thus, biases exist in these mutation detections and comparisons. Third, the deleterious mutations reported here are more likely to be harmful [9,23,25] but are still predictive in nature. Further research is needed on the plant fitness consequences of these predicted deleterious mutations on the assayed accessions. It is possible that the identified mutations accumulated in response to cold storage conditions may still be detrimental to plant fitness under future environmental conditions. An early study revealed that some phenotypic mutations were induced during storage in barley and pea seeds [5]. Fourth, many of the reported correlations of burden estimates with other mutation-related estimates or conservation-related accession features are statistically significant, but they explained only a relatively small proportion of the observed variances. Thus, caution is still warranted when interpreting these correlations for a mutation burden prediction. Fifth, this study considered only three conservation-related accession features. Other genebank operational procedures and conditions, such as seed drying conditions, seed exposure to changing temperatures, frequency of seed thawing and freezing for assessments, and seed aging during storage, may also be associated with the generation of deleterious mutations.

The findings reported here not only advance our knowledge on the accumulation of heterozygous deleterious mutations in conserved soybean germplasm through germplasm regenerations but also have some practical implications for germplasm management and conservation. First, the revealed mutation accumulation in conserved soybean germplasm provides additional support for the early notion that the genetic cost of deleterious mutations should be considered as a cost factor in genebank management practices with the goal to minimize the extent of mutation accumulation in conserved germplasm [6,61]. Inevitably, the original genetic profiles of conserved germplasm will change over time in conservation [11]. However, effective mitigating measures to minimize the genetic changes have not been developed yet and are currently missing in worldwide genebank operations [32,33]. Second, the genetic cost for plant germplasm conserved in genebanks will increase over long-term conservation, even with a low deleterious mutation rate such as 1.06 × 10^−8^ estimated for soybean germplasm [22], as germplasm regenerations, essential to maintain long-term germplasm survival and availability, will increase [62], providing more opportunities to accumulate deleterious mutations. Thus, minimizing germplasm regeneration cycles through a better viability prediction of conserved germplasm [63] should be a focus for genebank operations [62]. However, the current viability testing and prediction procedures used in genebanks are far from optimized to ensure effective germplasm viability monitoring [64,65]. Also, better regeneration practices with a large sample size should be applied, if feasible, to minimize the fixation of deleterious mutations from genetic drift. Third, more research is needed to investigate the processes of deleterious mutation accumulation in conserved germplasm, as the findings reported here may be specific to selfing plants and not general to outcrossing plant germplasm. For outcrossing species, genetic purging of heterozygous and/or homozygous deleterious mutations may differ during germplasm regeneration [6,51,66]. Even for selfing plant germplasm, the generality to other plant species of the revealed heterozygous mutation accumulation remains to be empirically determined. Also, the revealed association between sample origins and total mutation burden estimates (Figure S6A) suggests the need for more research to assess the generality of this association, as such a generality may inform genebank operations to minimize mutation accumulation in the germplasm from specific countries of origin. Moreover, the results of homozygous mutation accumulation (Figure 1C) were not fully expected based on the early simulations (see Figure 3B of Schoen et al. [6]). This line of mutational research will advance our understanding of mutation accumulation in conserved germplasm and support the long-term conservation of plant germplasm.

## 5. Conclusions

This seed-based RNA-Seq analysis identified 588 deleterious SNPs, which were widely distributed across 20 soybean chromosomes, mostly present in only a few samples, associated with diverse biological processes, and largely predicted to be weakly and mildly detrimental. The estimates of total and heterozygous mutation burdens were found to increase significantly with the number of conservation years since accession acquisition and the number of regeneration cycles, but the estimates of homozygous mutation burden were not associated with these two conservation-related accession features. These findings provide the first empirical evidence that total mutation burden increased mainly through the accumulation of heterozygous, rather than homozygous, deleterious mutations over soybean germplasm regenerations. This insight is useful for conducting informative assessments of deleterious mutation accumulation and enhancing the management and conservation of plant germplasm.

## Figures and Tables

**Table 1:** Results of annotating genetic variants detected in 190 soybean accessions and identifying deleterious SNPs.

Variant	Count/Proportion	Variant	Count/Proportion
***SNP calling and filtering***		Loss-of-function variant **	
Total SNPs without missing values	58,548	Total count	869
SNP annotation with VEP *		Proportion	0.015
Missense_variant (MV)	19,847	***SIFT analysis with CT*** ***	
Proportion of MV in total SNPs	0.339	SIFT-deleterious SNPs (SDS)	5912
Synonymous_variant (SV)	20,478	Proportion of SDS in total SNPs	0.101
Proportion of SV in total SNPs	0.350	Deleterious_low_confidence SNPs	941
Splice_acceptor_variant	45	Tolerated SNPs	34,076
Splice_donor_variant	50	***Deleterious SNPs by SIFT+RS***	
Stop_gained	450	SDS+RS-filtered SNPs (RSD)	588
Stop_lost	57	Proportion of RSD in total SNPs	0.010
Start_lost	45	Fixed RSD	1
Splice_region_variant	194	Proportion of fixed RSD in total SNPs	0.00002
Stop_retained_variant	28	Weakly deleterious with RS < 1	45
5_prime_UTR_variant	5354	Proportion of RSD	0.077
3_prime_UTR_variant	7068	Mildly deleterious with RS of 1–3	477
Non_coding_transcript_exon_variant	208	Proportion of RSD	0.811
Intron_variant	1131	Highly deleterious with RS > 3	66
Upstream_gene_variant	1584	Proportion of RSD	0.112
Downstream_gene_variant	863		
Intergenic_variant	1146		

* The most severe consequence class of VEP. ** Loss-of-function variants consist of those variants from the annotation classes of three “STOP_”, three “Splice_” and one “Start_lost”. *** SIFT-filtered with canonical transcripts (CT).

## Data Availability

The original RNA-Seq sequence data were deposited in NCBI’s SRA database under the BioProject ID PRJNA1140562. Some research outputs can be found in the Supplementary Materials.

## Supplementary Materials

The supporting information can be downloaded at https://doi.org/10.6084/m9.figshare.29374274 (accessed on 20 June 2025).
